# BGLE-YOLO: A Lightweight Model for Underwater Bio-Detection

**DOI:** 10.3390/s25051595

**Published:** 2025-03-05

**Authors:** Hua Zhao, Chao Xu, Jiaxing Chen, Zhexian Zhang, Xiang Wang

**Affiliations:** 1 School of Mathematical Sciences, Hebei Normal University, Shijiazhuang 050024, China; zhaohua@hebtu.edu.cn; 2 School of Engineering, Hebei Normal University, Shijiazhuang 050024, China; 17333921516@163.com (Z.Z.); wangxiang@hebtu.edu.cn (X.W.)

**Keywords:** underwater fish detection, BGLE-YOLO, YOLOv8, multi-scale feature fusion, lightweight model

## Abstract

Due to low contrast, chromatic aberration, and generally small objects in underwater environments, a new underwater fish detection model, BGLE-YOLO, is proposed to investigate automated methods dedicated to accurately detecting underwater objects in images. The model has small parameters and low computational effort and is suitable for edge devices. First, an efficient multi-scale convolutional EMC module is introduced to enhance the backbone network and capture the dynamic changes in targets in the underwater environment. Secondly, a global and local feature fusion module for small targets (BIG) is integrated into the neck network to preserve more feature information, reduce error information in higher-level features, and increase the model’s effectiveness in detecting small targets. Finally, to prevent the detection accuracy impact due to excessive lightweighting, the lightweight shared head (LSH) is constructed. The reparameterization technique further improves detection accuracy without additional parameters and computational cost. Experimental results of BGLE-YOLO on the underwater datasets DUO (Detection Underwater Objects) and RUOD (Real-World Underwater Object Detection) show that the model achieves the same accuracy as the benchmark model with an ultra-low computational cost of 6.2 GFLOPs and an ultra-low model parameter of 1.6 MB.

## 1. Introduction

**Figure 1:**
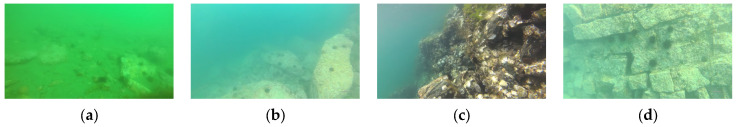
Images (**a**,**b**) describe underwater image features marked by low contrast and small targets, respectively, and (**c**,**d**) describe underwater image features marked by underwater blurring and color deviations due to various attenuations, respectively.

With the maturity of target detection technology on the ground, the development of underwater resource exploitation, marine ecological protection, and underwater robots has become a trend, and the vigorous growth of the marine economy is a major guarantee of national security [1]. Target detection is crucial for the protection and exploitation of seabed organisms as well as resource systems, and underwater image processing has been widely used in a variety of underwater scenarios, playing an important role in exploring underwater scenarios. Nevertheless, dynamic underwater environments make damaged underwater areas more complex and unpredictable, and the development of underwater robots requires underwater target detection algorithms with superior computational and storage capacity, which is exacerbated by the limited algorithmic storage and computational capacity at this stage. Secondly, underwater environments suffer from several problems such as low light, dense distribution of organisms, low contrast, and small object size (see Figure 1). Because of these challenges, underwater images suffer from blurred image boundaries and loss of texture information, making underwater target detection more difficult [2].

In target detection techniques, there are two categories of algorithms: two-stage algorithms and single-stage algorithms. Two-stage algorithms first extract the candidate frame based on the image and then do a quadratic correction based on the candidate region to obtain the detection point result. Common two-stage target detection algorithms: R-CNN [3], Fast R-CNN [4], and Faster R-CNN [5]. Single-stage target detection algorithms omit the extraction of candidate frames and directly classify and localize targets on the feature map. Typical single-stage target detection algorithms: SSD [6] and the YOLO (You Only Look Once) [7,8] series. Compared to the two-stage target detection algorithms, the single-stage target detection algorithms have lower detection accuracy, but their detection speed is fast, with relatively low computational overhead and good real-time performance. The YOLO series of target detectors are widely used in academic and industrial scenarios due to their high performance and efficiency, and they are proven to be a classical approach for first-level detection networks in practical applications. However, despite the YOLO algorithm’s strong performance in real-time target detection, its effectiveness is confined to particular application scenarios, such as dynamic environments underwater.

Underwater target detection differs from the ground environment in that the underwater environment is more complex, and the difficulty of creating and labeling datasets increases by orders of magnitude. Underwater targets are generally small, making them hard to capture and recognize, which inevitably results in decreased effectiveness of certain target detection algorithms. In addition to accuracy, computation, and parameterization are two indispensable components for porting target detection algorithms to underwater mobile devices. The development of detection algorithms suitable for underwater scenarios should be a combination of detection accuracy and practical applications. Recent research has been conducted to develop models specialized for underwater detection. Liu et al. [9] studied YWnet to resolve the problem related to small and blurred target sizes in underwater environments. Yeh et al. [10] introduced a lightweight algorithm for detecting underwater targets, which integrates a color conversion technique with a neural network to address the issue of color absorption. Nonetheless, these techniques often depend significantly on the natural quality of underwater images, and excessive enhancement may result in the loss of important image details, which can adversely affect detection accuracy. Existing methods mostly ignore significant computational overheads when addressing underwater environmental challenges, which hinders their practical deployment.

To address the above problems, Xu et al. [11] proposed the use of inverted residual blocks and group convolution [12] to extract deep features, which enables the feature extraction network to give priority to channels that contain a lot of information and discard insignificant ones, which simplifies the differentiation between perceptual targets and background information, further improves the accuracy of the model, and significantly reduces the memory footprint required for inference. Although it greatly decreases the memory needed for inference and improves accuracy, it occupies more computational consumption and parameter storage, limiting the storage and computational requirements for underwater image processing. Tang [13] explored the localization capabilities of small target segmentation and fuzzy target boundaries in medical image processing and found that the ability of the Global-to-Local Spatial Aggregation (GLSA) module to aggregate and represent both global and local spatial features provides significantly improved localization results for both large and small underwater targets, but its huge number of parameters potentially limits its use in resource-constrained scenarios. Chen [14] developed a streamlined backbone network using the Deformable Convolutional Network (DCN) v3 [15] to fuse feature maps from different scales and achieved a mAP@0.5 of 86.1% on the DUO dataset. Their research, however, added to the computational complexity and curtailed its application in scenarios with real-time and resource constraints. Detail-enhanced convolution (DEConv) [16] promotes feature learning and can integrate prior information to supplement the original information and enhance representation capabilities, but it is often not advisable to significantly reduce model parameters and computation at the expense of accuracy.

In order to optimize the above problems, the paper presents a BGLE-YOLOv8 model that is lightweight, building upon structural improvements in YOLOv8. The main points of the work are as follows:

1. To achieve a lighter YOLOv8 backbone network, the parameter count and computational load are designed to be lower than the original 3 × 3 convolution kernel for EMC convolution. Inheriting the grouping idea of Group Convolution, multi-scale feature information is extracted more efficiently by the backbone network during the feature extraction process.
2. Introducing the BIG module. The BIG module can reduce the error information generated in the high-level features as the detection depth increases. In the neck network, its local spatial detail information and global spatial semantic information extracted from the backbone part are able to perform fast and efficient multi-scale feature fusion.
3. The LSH module is introduced into the architecture because it references the fact that shared convolution can drastically lower the parameter count and enables the use of a Scale layer to scale the features when dealing with the problem of inconsistent underwater target scales detected by each detection head. Detail-enhanced convolution and group normalization are also designed to improve its detail-capturing ability and minimize the accuracy loss while keeping the number of detection head parameters and computational effort smaller.

The remainder of the paper is arranged as listed below. Section 2 presents the overall network structure as well as its individual components, fully exploring the role played by each group block in the overall network structure and the correlation between each module. Section 3 provides the experimental results, which fully confirm the validity of the proposed model through ablation and comparison experiments. Section 4 presents a discussion, summarizes the proposed model and its limitations, and suggests an outlook for the improvement of the subsequent benchmark model. Finally, Section 5 provides the research conclusion.

## 2. Materials and Methods

**Figure 2:**
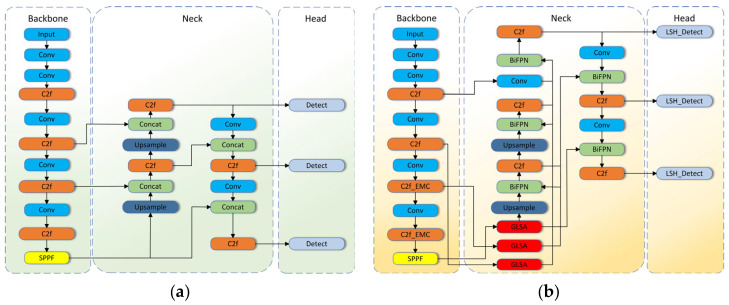
(**a**) The diagram illustrates the architecture of YOLOv8. (**b**) The diagram depicts the architecture of BGLE-YOLO. Compared to (**a**), (**b**) adds BiFPN network, GLSA attention block, EMC convolution, and LSH detection header to (**a**).

In this part, we plan to introduce the different modules of the model. Figure 2 displays the overall architecture of the baseline model and the model in this paper. The BGLE-YOLO model consists of the following parts: efficient multi-scale convolution EMC, BIG module, and lightweight detection head LSH.

### 2.1. Efficient Multi-Scale Convolution EMC

In the scope of underwater target detection, limited hardware resources and computing power make it tough to deploy convolutional neural networks on embedded systems or edge devices. This can usually be achieved through model compression or lightweight design. Deep convolutional neural networks [17,18] are usually composed of a large number of convolutions, which results in substantial computation, parameters, and model complexity, resulting in slow model inference speed.

Han [19] proposed the Ghost module to decompose a convolutional layer into two smaller convolutional layers. One of the convolutional layers is called the ghost convolutional layer, which only uses a small part of the channels of the original convolutional layer to calculate the output, while the other convolutional layer is called the residual convolutional layer, which uses the remaining channels to calculate the output. This decomposition technique can significantly decrease the computational load and number of parameters while preserving the network’s accuracy. Although Ghostnet has achieved good performance, it does not fully consider the redundancy between feature maps, assuming that these redundant feature maps need to be fully removed to provide a comprehensive insight into the input data. MobileNet [20] uses deep convolution to perform convolutional feature extraction operations on the layer-wise channel count, but the 1 × 1 convolutional layer of the remaining fusion channel still takes up a considerable amount of memory and GFLOPs.

**Figure 3:**
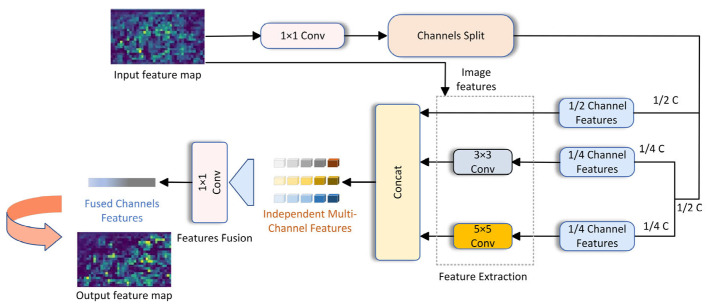
The structures of EMC are presented. The input feature map is channelized and then fused into an output feature map by independent multi-channel features.

Building on previous research, this paper suggests enhancements to the network architecture, specifically targeting the underwater environment. The improved model is capable of reducing the parameter count and calculations based on previous work and maintaining detection accuracy and adaptability. These feature maps are generated on a single feature channel, with each channel’s information being independent from one another. Specifically, each channel of the feature map possesses its own convolution kernel, which operates exclusively on that channel. Half of the feature maps input into the channel are not convolved, and the other half are convolved with a kernel size of 3 and a kernel size of 5. Finally, the features of each channel after the convolution operation can be fused through a point-by-point convolution. Figure 3 illustrates the EMC network structure. Based on this principle, the correlation and redundancy between redundant feature maps can be fully explained, and the problem of significantly reducing model parameters and calculations while retaining redundant feature maps and effectively improving the detection accuracy of underwater targets can be solved.

When EMC replaces the original convolution and integrates it into the YOLO framework, it has several advantages, making it particularly suitable for underwater object detection. This module enables the model to pay more attention to the essential visual features for object detection by fully maintaining the redundancy and correlation among feature maps.

### 2.2. BIG

**Figure 4:**
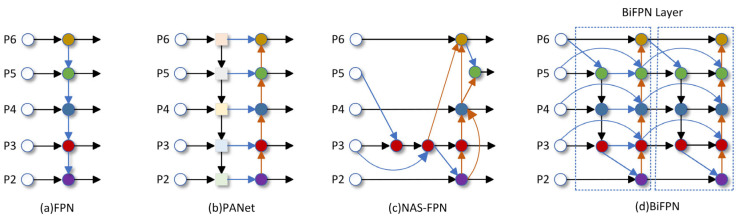
The structures of FPN, PANet, NAS-FPN, and BiFPN.

A major challenge in underwater fish detection is to be able to highlight key information related to the target while removing irrelevant features. Given that underwater targets are generally small and occlude each other, existing target detection algorithms struggle to effectively detect them. Traditional feature pyramid networks (FPNs) [21] are limited by unidirectional information flow, thus the Path Aggregation Network (PANet) [22] incorporates an extra bottom-up path aggregation network. To explore a more effective cross-scale feature network topology within the PANet, work [23] revealed that the search process consumes thousands of GPU hours, and the resulting network is irregular and challenging to interpret or modify. Inspired by the above works, the bi-directional feature pyramid network (BiFPN) [24] achieves better accuracy and fewer parameters and calculations than the PANet, FPN, and neural architecture search (NAS-FPN). The optimization methods of BiFPNs for cross-scale connections are as follows: deleting nodes with only one input because there is no feature fusion process for one input node. Secondly, the original input and input nodes are positioned at an identical level, allowing for more feature fusion without significantly increasing computational cost. Lastly, each bidirectional path is regarded as a feature network layer and repeated several times for more advanced feature fusion. For the feature fusion network, the BiFPN can highlight the key information of underwater targets and remove irrelevant features, truly achieving a lightweight model and better accuracy under resource constraints. The structures of the FPN, PANet, NAS-FPN, and BiFPN are shown in Figure 4.

**Figure 5:**
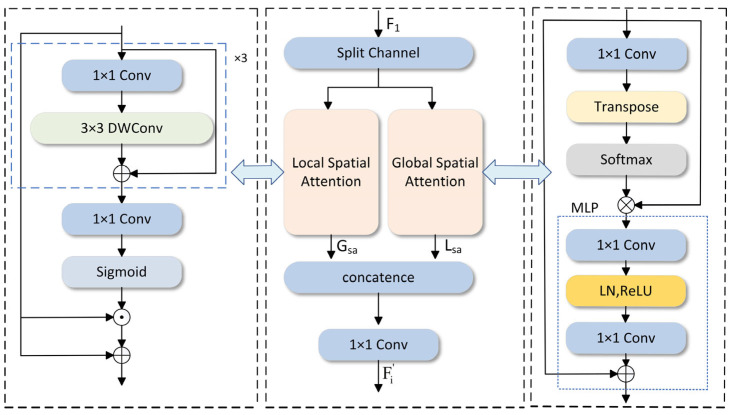
Introduction to the Global-to-Local Spatial Aggregation (GLSA) module.

Detecting small-scale targets in medical imaging and identifying defects in aircraft and underwater fish images are specific detection tasks. The specific target distribution is denser and more blurred, and it is hard to apply the BiFPN framework directly to achieve better detection results in complex underwater environments. To address this problem, a method BIG based on BIFPN-weighted bidirectional feature fusion is proposed, replacing the three convolution blocks that adjust the channel count using the GLSA attention module (as depicted in Figure 5).

The GLSA module is composed of two parts: the GSA module and the LSA module. GLSA enhances the attention mechanism to refine target-related information and diminish irrelevant details. To simultaneously capture global and local spatial features, it combines two independent local and global attention units. The global attention module highlights the long-range spatial relationships between pixels, complementing the local spatial attention. Meanwhile, the local attention module efficiently extracts the local features of interest areas in underwater fish images, such as small target objects, within the spatial dimension of a given feature map. Under the common strategy of weighted feature fusion and global–local spatial attention, the ability to quickly and efficiently fuse multi-scale features, covering local spatial details and global spatial semantic information extracted from the backbone, has been significantly enhanced. This dual-stream design effectively maintains local and non-local modeling, using separate channels to balance accuracy and computational resources. This design increases the mAP@0.5:0.95 of the model by 1.1%, reduces the number of parameters by 32%, and reduces the number of calculations by 6%, which strengthens the model’s detection accuracy and robustness. The input feature map is represented by $\left\{F_{i}\left|i\in 2,3,4\right.\right\}$; it uses separation channels to evenly divide the 64 channels of $F_{i}$ into two sets of feature maps, as follows: $F_{i}^{1},F_{i}^{2}(i\in 2,3,4)$. The two sets of feature maps are, respectively, fed into the Global Spatial Attention (GSA) module and the Local Spatial Attention (LSA) module. The two attention units are finally cascaded together and then output through a 1 × 1 convolution layer $F_{i}^{\prime }$. The whole process is as follows:

(1) $$F_{i}^{1},F_{i}^{2}=Split(F_{i})$$

(2) $$F_{i}^{\prime }=C_{1\times 1}(Concat(G_{sa}(F_{i}^{1}),L_{sa}(F_{i}^{2})))$$

Here, $G_{sa}$ represents the global spatial attention mechanism and $L_{sa}$ represents the local spatial attention mechanism. $F_{i}^{\prime }\in {\mathbb{R}}^{\frac{H}{8}\times \frac{W}{8}\times 32}$ indicates the output of the GLSA module. The global spatial attention GSA and local spatial attention LSA are introduced below.

#### 2.2.1. GSA Module

Underwater images are often partially blurred, color-shifted, and low-contrast, making it difficult to detect fish based on partially missing image information. Therefore, the long-range interactions between each pixel in a single image are particularly important. The work [25] proposed that the use of a self-attention mechanism and query-independent global context modeling can model the long-distance dependencies in machine translation well. Ref. [26] used the self-attention mechanism to simulate pixel-level pairwise relationships. When performing underwater image detection, the global attention module can perform global long-distance modeling based on the relationship between objects and pixels in object detection. Based on the findings from [27] that long-distance interactions can make object detection more powerful, a global spatial attention map can be generated. The process is as follows:

(3) $$Att_{G}(F_{i}^{1})=softmax(Transpose(C_{1\times 1}(F_{i}^{1})))$$

(4) $$G_{sa}(F_{i}^{1})=MLP(Att_{G}(F_{i}^{1})⊗F_{i}^{1})+F_{i}^{1}$$

$Att_{G}\left(\cdot \right)$ indicates attention operation, while $C_{1\times 1}$ indicates a 1 × 1 convolution. ⊗ indicates matrix multiplication. $MLP\left(\cdot \right)$ is composed of two fully connected layers, a ReLU activation function and a normalization layer. The global spatial attention mechanism emphasizes the long-range relationship between objects and pixels in a single underwater image throughout the process.

#### 2.2.2. LSA Module

Detecting underwater targets is a crucial technology for the advancement of intelligent underwater robots. However, small and dense targets still face severe challenges in target detection. The work [28] integrates the transformer self-attention mechanism and the coordinate attention mechanism with the adaptive histogram equalization algorithm’s image enhancement technique to greatly improve underwater target feature extraction and reduce the number of blurred bounding boxes. Although it effectively reduces the accuracy loss caused by smaller targets, it increases the computational cost and parameter amount compared to the baseline model, which is very difficult to deploy on edge or embedded devices. The LSA module can effectively extract local features of the region of interest, such as small objects, on a given feature map. The response calculation of LSA is given below:

(5) $$Att_{L}(F_{i}^{2})=\sigma (C_{1\times 1}(F_{c}(F_{i}^{2}))+F_{i}^{2}))$$

(6) $$L_{sa}=Att_{L}(F_{i}^{2})⊙F_{i}^{2}+F_{i}^{2}$$

Among them, $F_{c}\left(\cdot \right)$ represents a sequence of three 1 × 1 convolutional layers followed by a 3 × 3 depth-wise separable convolutional layer, $Att_{L}\left(\cdot \right)$ is the local attention operation, $\sigma \left(\cdot \right)$ is the Sigmoid function, and ⊙ is the point-by-point multiplication. The design of LSA enables this module to reduce the computational load and the number of parameters compared to standard convolution, and it can more effectively aggregate local spatial information through cascading and residual connections.

### 2.3. LSH

**Figure 6:**
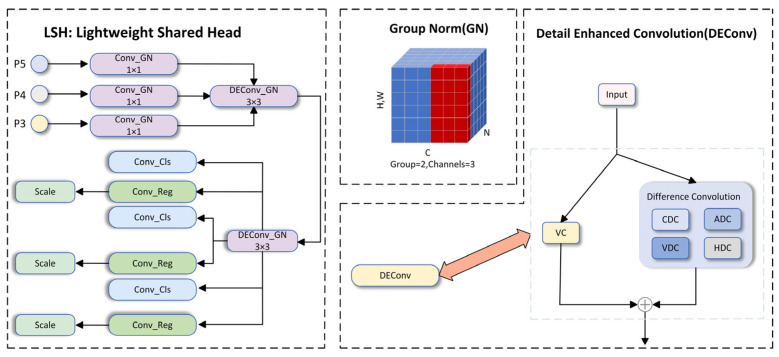
Convolution in LSH consists of the group normalized GN convolution and the group normalized detail-enhanced convolution DEConv. The red pixels are normalized using the same mean and variance, which are calculated by combining the values of these pixels.

The detection head network is generally responsible for localization and classification detection. In [29,30], using shared convolutions to share heads across different feature levels not only enhances the efficiency of detector parameters but also effectively reduces the computational burden of the head network, ultimately giving the target detection head better representation capabilities. In complex underwater environments, shooting equipment often needs to simulate objects of different distances and sizes for shooting. The captured images need to be rescaled, using the scale layer and scaling the features of different sizes and distances. Inspired by detail enhancement convolution (DEConv) and group normalization (GN) [31], a lightweight detection head LSH is proposed. Figure 6 illustrates the LSH structure. The following introduces the two main components of LSH to illustrate the irreplaceable role of group normalization (GN) and detail enhancement convolution (DEConv) in the detection head.

#### 2.3.1. Group Normalization

Batch normalization (BN) is a pivotal technology in deep learning development, facilitating the training of various detection networks. However, as normalization introduces problems along the batch size, the error of BN becomes uncontrollable. For underwater target detection tasks and training some larger models, deployment issues are limited. Group normalization (GN), serving as an alternative to batch normalization, achieves a better balance between accuracy and parameters. For different batch sizes, GN’s error rate is 10.6% lower than BN. GN partitions the input channels into groups and computes the normalized mean and variance for each group individually. The specific calculation is as follows:

(7) $${\hat{x}}_{i}=\frac{1}{\sigma_{i}}(x_{i}-\mu_{i})$$

The *x* above is the feature calculated by the layer. In the case of 2D images, $i=(i_{N},i_{C},i_{H},i_{W})$ is a 4D vector indexing features in the order (*N*, *C*, *H*, *W*), where *N* is the batch axis, *C* is the channel axis, and *H* and *W* are the spatial height and width axes. Among them, $\mu_{i}$ and $\sigma_{i}$ can be obtained by the following formula:

(8) $$\mu_{i}=\frac{1}{m}\sum_{k\in S_{i}}x_{k}$$

(9) $$\sigma_{i}=\sqrt{\frac{1}{m}\sum_{k\in S_{i}}{\left(x_{k}-\mu_{i}\right)}^{2}+\varepsilon }$$

where ε is a small constant, $S_{i}$ is the set of pixels for calculating the average and standard value, and m is the size of the set. Batch normalization (BN) and Group normalization (GN) also differ greatly in how the set $S_{i}$ is defined. Here, we mainly introduce the definition of group normalization. The set $S_{i}$ definition of GN is as follows:

(10) $$S_{i}=\{k\left|k_{N}=i_{N},\left\lfloor \frac{k_{C}}{C/G}\right\rfloor =\left\lfloor \frac{i_{C}}{C/G}\right\rfloor \right.\}$$

Here, *G* represents the number of groups, which defaults to 32. *C*/*G* denotes the number of channels per group. $\left\lfloor \cdot \right\rfloor$ is the rounding operation, while $\left\lfloor \frac{k_{C}}{C/G}\right\rfloor =\left\lfloor \frac{i_{C}}{C/G}\right\rfloor$ shows that index *i* and *k* belong to the same group of channels. Here, it is assumed that each group of channels is arranged sequentially along the *C* axis, and GN calculates the σ and μ along the (H, W) axis and a set of *C*/*G* channels. When *G* = 2, that is, there are 2 groups, each with three channels. The calculation method of GN is shown in Figure 6.

#### 2.3.2. Detail Enhancement Convolution

Some previous works [32] proposed that images taken in blurry conditions often experience visual quality degradation in contrast or color distortion, which leads to performance degradation of some convolution neural network (CNN)-based methods. Specifically, the model’s robustness and generalization ability are enhanced by detail enhancement convolution through the integration of prior information into standard convolution layers. Then, DEConv is converted into a vanilla convolution equivalent through the re-parameterization technique. Through such a process, the parameters and computational cost of the model are not increased. In the detail enhancement convolution, five convolution layers are used: vanilla convolution (VC), center difference convolution (CDC), angle difference convolution (ADC), vertical difference convolution (VDC), and horizontal difference convolution (HDC). They are integrated into the convolutional layers with traditional local descriptors such as Sobel [33]. Figure 6 displays the DEConv structure. To mitigate the increase in parameters and inference time resulting from deploying five convolutional layers, given the input feature $F_{in}$, DEConv uses re-parameterization technology to output $F_{out}$ to the ordinary convolutional layer with the same parameters and inference time. The calculation of DEConv is expressed as (11).

(11) $$F_{out}=DEConv(F_{in})=\sum_{i=1}^{5}F_{in}\times K_{i}=F_{in}\times (\sum_{i=1}^{5}K_{i})=F_{in}\times K_{cvt}$$

Among them, DEConv(⋅) denotes the DEConv operation, while $K_{i=1:5}$ signifies the cores of VC, CDC, ADC, HDC, and VDC, respectively. $K_{i}$ indicates the convolution operation, and $K_{cvt}$ stands for the transformed kernel that concatenates parallel convolutions.

## 3. Experiments and Discussion

### 3.1. Experimental Environment and Configuration

In this paper, the DUO dataset and the RUOD dataset are used for model implementation. The DUO dataset serves as a resource for underwater object detection in robotic harvesting [34]. This dataset collects a variety of underwater images and is equipped with more accurate annotations and a variety of challenging underwater environment images. The dataset covers four categories: holothurian, echinus, scallops, and starfish, with a total of 7782 images. In this dataset, the training set comprises 6671 images, while the test set consists of 1111 images.

The RUOD dataset [35] includes 14,000 high-resolution images and 74,903 labeled instances. Moreover, the dataset includes various environmental challenges, including haze-like effects, color bias, and light interference. It is the first generalized dataset for underwater object detection, taking into account marine objects and environmental challenges. The dataset covers ten categories: holothurian, echinus, scallop, starfish, fish, corals, diver, cuttlefish, turtle, and jellyfish. The dataset consists of 9800 training images and 4200 testing and evaluation images.

The experiments were carried out on a Linux system equipped with an NVIDIA GeForce RTX 4090D GPU and an Intel i9-14900KF CPU. The BGLE-YOLO model was developed using the PyTorch 2.4.1 deep learning framework. The software environment included CUDA 12.1 and Python 3.9. The model was trained for 300 epochs with a learning rate of 0.01. Multiple experiments were conducted through iterative parameter tuning, and finally, some key hyperparameters were standardized, and all experiments were performed with the same configuration displayed in Table 1. In order to speed up the training, the batch size was set to 32. In addition, a cache was activated for this experiment and 8 work processes were configured to improve the training efficiency. Table 1 presents the detailed parameters of the experimental setup.

### 3.2. Evaluation Metrics

In order to evaluate the performance of the BGLE-YOLO model, this paper utilizes the following evaluation metrics: Precision, AP, mAP@0.5, and mAP@0.5:0.95. True positive (TP) denotes the count of samples accurately predicted as positive, while false positive (FP) indicates the number of samples predicted as positive but incorrectly so; true negative (TN) represents the count of samples accurately predicted as negative, and false negative (FN) indicates the number of samples incorrectly predicted as negative.

*Precision*: *Precision* is the ratio of the positive class predicted by the model to the positive class predicted. The calculation formula is as follows:

(12) $$Precision=\frac{TP}{TP+FP}$$

Average Precision (*AP*): The *AP* value is equivalent to the area beneath the precision-recall curve and the coordinate axis. The calculation formula is as follows:

(13) $$AP=\int_{0}^{1}P(R)dR$$

Mean Average Precision (*mAP*): The *mAP* value is calculated by dividing the sum of the average precision (*AP*) across all classes in the dataset by the total number of classes. The formula for this calculation is as follows:

(14) $$mAP=\frac{1}{N}\sum_{i=1}^{N}AP_{i}$$

In addition to the above evaluation indicators, this study also examines the complexity of the model, namely the number of model parameters and the amount of computation. Therefore, the parameters and GFLOP metrics are introduced to comprehensively evaluate the model’s adaptability to edge devices and hardware platforms.

### 3.3. Ablation Experiments

#### 3.3.1. Prediction Problem of Group Normalization in LSH

As mentioned in the previous section, the experiment replaced batch normalization (BN) in the LSH detection head with group normalization (GN). It can be seen from the experiment that the introduction of group normalization (GN) is very necessary. Table 2 presents the outcomes of the ablation experiment on the DUO dataset. When the detection head does not introduce detail enhancement convolution (DEConv), the mAP@0.5 of BGLE-YOLO with group normalization (GN) is 0.2% higher than that without GN. When detail enhancement convolution (DEConv) is introduced, the mAP@0.5 and mAP@0.5:0.95 of BGLE-YOLO with group normalization (GN) are 0.3% and 0.4% higher than those without GN, respectively. This shows that using GN alone can slightly enhance the detection performance of BGLE-YOLO without increasing the complexity of BGLE-YOLO while combining GN with DEConv can significantly enhance the classification and positioning capabilities of the LSH. The overall experiment proves that group normalization, as a substitute for batch normalization, does achieve better detection results in terms of accuracy.

Table 3 presents the ablation experiment results on the RUOD dataset. When the detection head does not introduce detail enhancement convolution (DEConv), the mAP@0.5 and mAP@0.5:0.95 of BGLE-YOLO after the introduction of group normalization (GN) are 1.4% and 2.4% higher than the mAP@0.5 and mAP@0.5:0.95 without GN. When LSH introduces detail enhancement convolution (DEConv), the mAP@0.5 of BGLE-YOLO after the introduction of GN is 0.1% higher than the mAP@0.5 without GN. This shows that regardless of whether DEConv is introduced or not, the introduction of GN can effectively enhance the accuracy of BGLE-YOLO.

#### 3.3.2. Performance Comparison of DEConv at Different Positions in LSH

In the LSH detection head, the classification convolution and regression convolution learned by the shared convolution output have the same target, which often needs to consider the scale of different targets. However, the classification convolution’s output parameters are shared regardless of target size, and the scale layer is unnecessary for feature scaling. To mitigate the problem of inconsistent scale in the detection head’s detection target, the parameters of the regression convolution need to scale the features. From the previous section, we know that using group normalization achieves better detection results than batch normalization. Here, the shared convolution parameters are displayed by changing the replacement position of the detail enhancement convolution in the LSH detection head. For convenience, all cases in this paper use the group normalization (GN) with better performance by default. At the same time, the convolution layer using the shared convolution parameters of DEConv (two groups of normalized convolutions with a convolution kernel size of 3 × 3, as shown in Figure 5) is denoted as RPC, and the convolution layer using the non-shared convolution parameters of DEConv is denoted as LPC (three groups of normalized convolutions with a convolution kernel size of 1 × 1, as shown in Figure 6). Then, the performance comparison before and after using detail enhancement convolution under different convolution layer types is presented, and Table 4 is formed.

Table 4 shows the ablation experiments with DEConv considering its position on the DUO dataset. "√" means add this module. Compared with not introducing DEConv anywhere, introducing DEConv into three 1 × 1 groups of normalized convolutions alone increases mAP@0.5 and mAP@0.5:0.95 by 1.2% and 0.8%, respectively, and the number of parameters and the calculation amount increase by 6% and 8%, respectively. Compared with introducing DEConv into three 1 × 1 groups of normalized convolutions alone, introducing DEConv into two 3 × 3 groups of normalized convolutions alone increases mAP@0.5 by 0.3%, and its number of parameters and calculation amount decrease by 6% and 8%, respectively. When DEConv is introduced into three 1 × 1 groups of normalized convolutions and two 3 × 3 groups of normalized convolutions at the same time, the accuracy, number of parameters, and level of calculation are all worse than when introducing DEConv into the two types of convolutions separately. It can be seen that when all convolutions are introduced with group normalization, introducing DEConv into two 3 × 3 convolutions alone can achieve optimal accuracy while minimizing the number of parameters and computations.

Table 5 shows the ablation experiments on the position where DEConv is added on the RUOD dataset. Compared with DEConv not introduced at any position, the introduction of DEConv in three groups of 1 × 1 normalized group convolutions alone reduces mAP@0.5 and mAP@0.5:0.95 by 0.1%. The introduction of DEConv in two groups of 3 × 3 normalized convolutions alone increases mAP@0.5 by 0.1% compared to not introducing DEConv at any position. When DEConv is added to two positions at the same time, the accuracy, parameters, and computational complexity are worse than adding DEConv alone or not introducing DEConv at any position. This shows that the combination of RPC and GN can better optimize the detection accuracy of BGLE-YOLO and effectively reduce parameters and computational complexity.

#### 3.3.3. Performance Comparison of BGLE-YOLO Components on the DUO and RUOD Datasets

As indicated in Table 6, the performance of each improved component of the BGLE-YOLO model is assessed. These improved components include (1) the EMC module, which fully retains the redundancy and correlation between feature maps to make the model more lightweight, (2) the BIG feature fusion module, which integrates the two attention mechanisms of the BiFPN structure and the GLSA module, and (3) the LSH detection head. To maintain the reliability and transparency of the experimental results, the experiment uses mAP@0.5 and mAP@0.5:0.95 as well as the model parameters and the model calculation GFLOPs as the main indicators of performance evaluation.

Relative to the baseline model, incorporating the EMC structure enhances model accuracy, with mAP@0.5:0.95 rising to 65.5%, which demonstrates the EMC module’s crucial role in multi-scale feature extraction. When the BIG module is introduced alone, all indicators are significantly improved, especially the accuracy and mAP@0.5:0.95. This shows that the BIG module plays an indispensable role in enhancing the performance of neck network feature fusion and also enables the model to achieve better lightweight effects while maintaining accuracy. When the LSH detection head is introduced, the lowest parameter effect is achieved while the model accuracy is consistent with the baseline model.

In addition, when the EMC and BIG modules are combined, the model performance is significantly better, the overall mAP@0.5:0.95 increases by 0.7%, and the number of parameters of the model is reduced by 33.3% compared to the baseline model. When the EMC, BIG, and LSH modules are incorporated simultaneously, the model’s overall performance is optimized. While the accuracy is the same as the benchmark model, the calculation amount and parameter amount are reduced by 24% and 46%, respectively. The BGLE-YOLO model is lightweight and has robust feature extraction and fusion capabilities, as indicated by experimental results, which meet the goals of detection tasks in complex underwater environments.

As indicated in Table 7, the EMC module slightly lowers the model’s computational load and parameter count while boosting accuracy by 0.2%, relative to the baseline model. When the BIG module is introduced alone, the model reduces the number of parameters by 29% and the amount of calculation by 6% while maintaining accuracy, indicating that the BIG module plays an indispensable role in achieving the lightweight effect of the model. After the LSH detection head is introduced alone, the model is superior to the baseline model in terms of accuracy, number of parameters, and amount of calculation, indicating that DEConv and GN can effectively achieve the lightweight effect of the model under the design of shared parameters.

In addition, when the EMC and BIG modules are used in combination, the model accuracy is slightly worse than that of the baseline model. In the combination of BIG and LSH, the model achieves a relatively ideal parameter amount and calculation level. When the EMC, BIG, and LSH modules are introduced together, the model’s overall performance is optimized. With similar accuracy, the model’s parameter and calculation amounts are reduced by 46% and 24%, respectively. This fully demonstrates that the proposed model can significantly reduce the parameter level and calculation burden while taking into account the accuracy.

### 3.4. Comparative Experiment

#### 3.4.1. Compared with the Traditional Lightweight YOLO Series

To emphasize the superior performance of BGLE-YOLO introduced in this paper, we first compare it with the traditional lightweight YOLO series. Table 8 displays the detailed data.

Based on the data in Table 8, it can be concluded that BGLE-YOLO highlights its superior performance under lightweight requirements. Although its accuracy and mAP@0.5 are slightly lower than those of YOLOv7-tiny, its mAP@0.5:0.95 is much higher than that of YOLOv7-tiny. In terms of computational efficiency, BGLE-YOLO has only 1.63 million parameters and 6.2 GFLOPs, much lower than YOLOv7-tiny (6.01 million, 13 GFLOPs).

As indicated in Table 9, BGLE-YOLO achieves comparable accuracy to other YOLO series models with fewer parameters and computational complexity. Although YOLOv8 and YOLOv11 are slightly higher than BGLE-YOLO in terms of mAP@0.5 and mAP@0.5:0.95, their parameters and computational complexity are higher than BGLE-YOLO, which does not meet the lightweight requirements. This indicates that the model proposed in this article is more suitable for subsequent deployment for edge devices.

**Figure 7:**
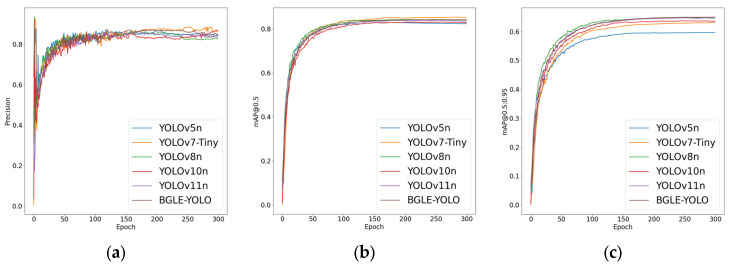
Comparison plots of the YOLO family of algorithms on the DUO dataset. (**a**) Accuracy comparison plot; (**b**) mAP@0.5 comparison plot; (**c**) mAP@0.5:0.95 comparison plot.

**Figure 8:**
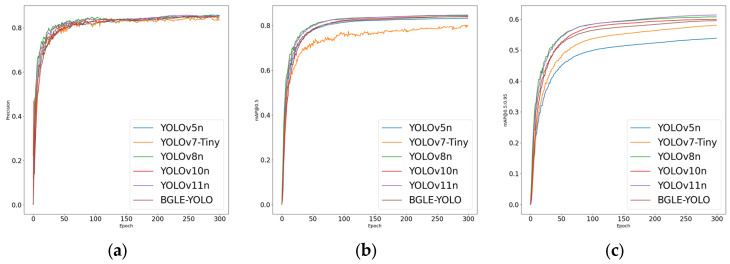
Comparison plots of the YOLO family of algorithms on the RUOD dataset. (**a**) Accuracy comparison plot; (**b**) mAP@0.5 comparison plot; (**c**) mAP@0.5:0.95 comparison plot.

In addition to the YOLOv7-tiny model, the comparison results with other models show that BGLE-YOLO has obvious advantages in the above parameter indicators (as shown in Figure 7 and Figure 8). BGLE-YOLO’s low computational complexity and simple design make it ideal for running on devices with limited computing power, like embedded systems, edge devices, etc.

#### 3.4.2. A Brief Comparison to Other Family Models

**Figure 9:**
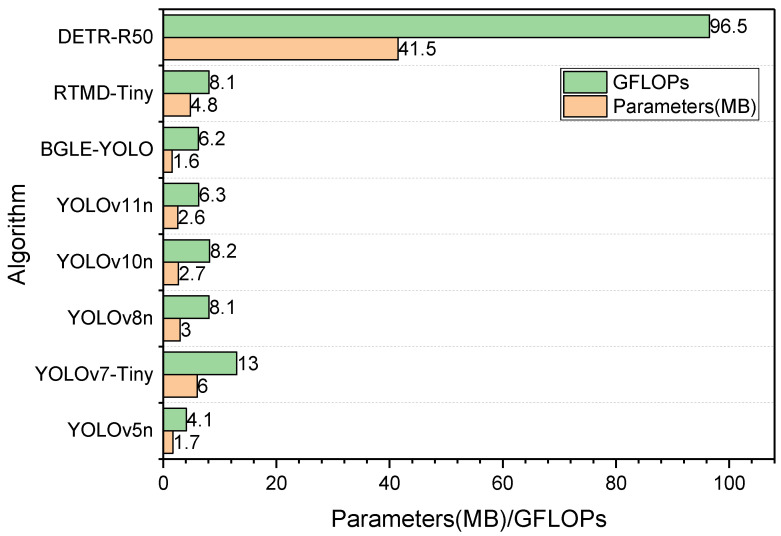
Comparison of parameters as well as computational effort of different models.

To further emphasize the lightweight advantages of the BGLE-YOLO introduced in this paper, this section compares the YOLO family as well as other models with the BGLE-YOLO (see Figure 9). Since the number of parameters and computation amount of BGLE-YOLO tested on the two datasets are almost the same, Figure 9 is based on the indicators of the proposed model tested on the DUO dataset. In order to better explore the relationship between the model parameters, computational complexity, and accuracy, this paper selects RTMD-Tiny as another model whose parameters and computational complexity are almost the same as those of the YOLO series models, and the experiment adds another model with huge parameters and computational complexity to compare the parameters and computational complexity with the remaining models. The detailed data are presented in Table 10 and Table 11.

Compared with other models outside the YOLO series, BGLE-YOLO achieves higher accuracy levels with fewer parameters and computational costs on both the DUO and RUOD datasets. On the DUO dataset, RTMD-Tiny requires 200% more parameters and 23% higher computational costs than the proposed model yet only achieves marginal improvements of 1.4% in mAP@0.5 and 1.5% in mAP@0.5:0.95 metrics. This outcome contradicts the lightweight design objectives emphasized in the paper. On the RUOD dataset, DETR-R50 requires 2400% more parameters than BGLE-YOLO, and the computational cost is 1300% higher. With a high computational complexity and parameter count, mAP@0.5 is only improved by 1.4%, and mAP@0.5:0.95 is 0.2% lower than BGLE-YOLO. Obviously, the model proposed in this paper is far superior to DETR-R50 in overall performance.

### 3.5. Visualization of Model Detection Effects on the DUO Dataset

**Figure 10:**
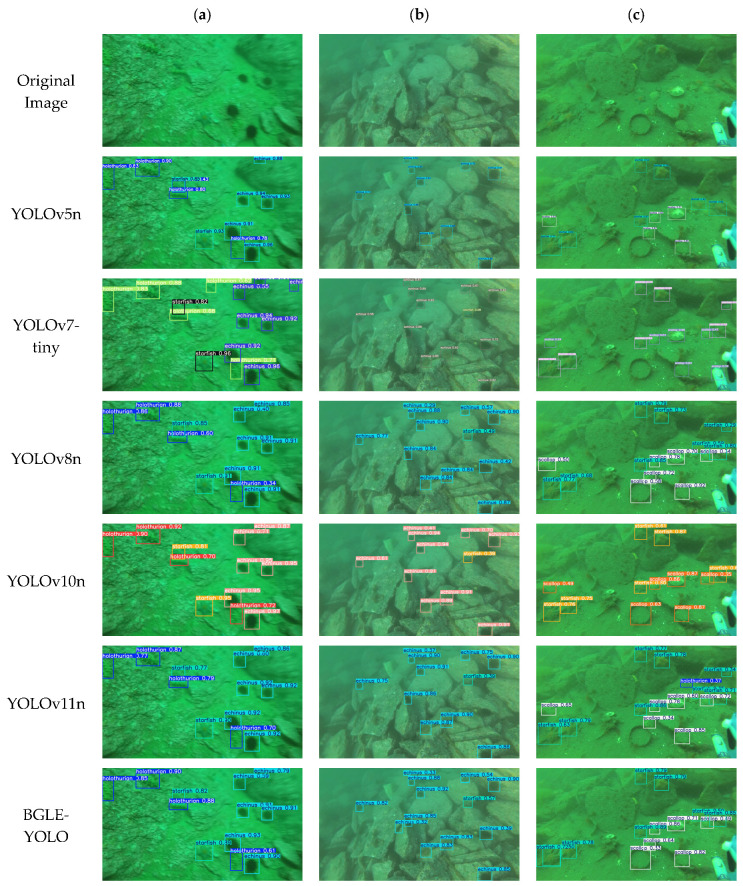
Qualitative comparison of the detection performance of the YOLO series of models, (**a**–**c**) showing the detection results for four categories in the DUO dataset.

To highlight the detection performance of the BGLE-YOLO model in underwater target recognition and modeling, we chose three images from different scenes in the DUO dataset to qualitatively analyze the various models. In Figure 10c, except for the BGLE-YOLO model proposed in this paper, all the other models have missed detection, which indicates that the other models perform poorly in feature extraction. Although the accuracy of this paper’s model is the same as that of YOLOv8n, the EMC module in BGLE-YOLO can fully account for the redundancy and correlation between feature maps, and the BIG module can fully integrate multi-scale features to mitigate leakage and false alarms.

### 3.6. Visualization of Model Detection Effects on the RUOD Dataset

**Figure 11:**
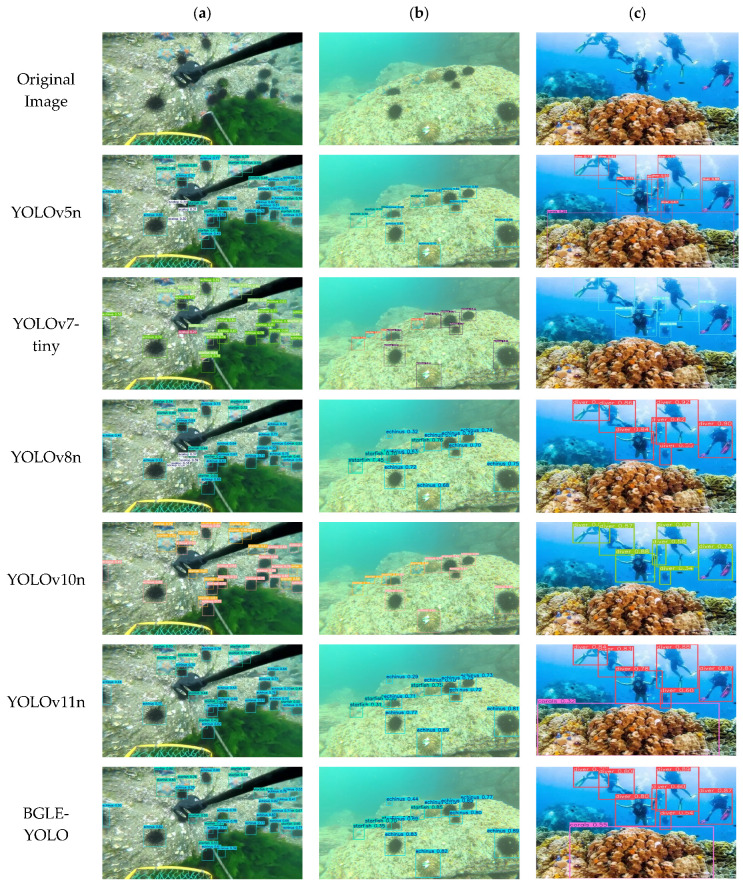
Qualitative comparison of the detection performance of the YOLO series of models, (**a**–**c**) showing the detection results for four categories in the RUOD dataset.

To emphasize the detection performance of the BGLE-YOLO model in underwater target recognition and modeling, we selected three images of different scenes in the RUOD dataset to conduct a qualitative analysis of each model. Also, in Figure 11c, except for the BGLE-YOLO model and YOLOv11 proposed in this paper, other models missed the detection of corals, which shows that other models are prone to miss key details when extracting small target features. In Figure 11a, except for YOLOv5 and YOLOv8, other models have different degrees of missed detection when detecting scallops. This shows that factors such as multi-target mutual occlusion and small objects have formed certain obstacles to the detection of marine life. Future work aims to overcome these problems based on lightweight design.

## 4. Discussion

### 4.1. Findings

This paper proposes a novel underwater fish detection method called BGLE-YOLO. It retains redundant feature maps while strengthening the correlation between feature maps by introducing EMC. Incorporating the EMC module alone can greatly decrease the parameters and computations of BGLE-YOLO. The BIG module is designed within the feature fusion network to enhance the long-range spatial relationships between pixels and effectively extract local features of interest in small underwater fish images. GN (group normalization) and DEConv (detail enhancement convolution) in the LSH module are crucial for the training of underwater target detection tasks, the balance between accuracy and parameters, and the problem of visual quality degradation in capturing images in terms of contrast or color distortion.

Ablation experiments conducted systematically verify the effectiveness of each innovative module. According to the DUO dataset ablation experiment, after the introduction of the EMC module, the mAP@0.5:0.95 of the model reached 65.5%, and the number of parameters and computational complexity were reduced by 9% and 6%, respectively. After incorporating BIG, mAP@0.5:0.95 rose to 65.7%, computational complexity dropped by 0.3GFLOPs, and the number of parameters was reduced by 21%, indicating that BIG played a key role in capturing global and local spatial features and optimizing convolution kernel parameters. Finally, the group normalization (GN) and detail enhancement convolution (DEConv) submodules in the LSH module significantly improved the performance of BGLE-YOLO. Activating the RPC position embedding DEConv and combining it with GN achieved the best accuracy, with mAP@0.5 and mAP@0.5:0.95 reaching 84.2% and 65%, respectively, and maintained the lowest level of computational complexity and parameters, which were reduced by 47% and 23%, respectively. In the ablation experiment of the RUOD dataset, BGLE-YOLO also showed a strong lightweight advantage. After adding EMC, the accuracy of the model increased to 85.4%, and the parameter level and calculation amount were reduced by 10% and 6%, respectively. With the introduction of the BIG and LSH modules, the mAP@0.5 of the model reached 84.1, and the number of parameters and calculation amount were lowered by 47% and 23%, respectively. The experiments above fully confirmed the effectiveness of these modules in enhancing model performance and reducing parameters and calculations.

### 4.2. Limitations and Future Works

Despite significant performance improvements on the RUOD and DUO datasets, BGLE-YOLO still faces some challenges. First, the accuracy of the model is equal to or slightly lower than the baseline model on both the RUOD and DUO datasets and higher accuracy may be required in some practical application scenarios. In addition, the generalization ability and robustness of BGLE-YOLO need to be further verified, and the model should be made more applicable to other datasets. Finally, with the development of underwater vision technology, more complex underwater environments come one after another, such as turbidity, fog effects, and color deviation. The subsequent series of work should focus on improving accuracy as much as possible with lower model parameters and computational burden. In addition, sufficient experiments should be combined with embedded or edge devices to verify effectiveness in different environments.

## 5. Conclusions

This research presents a novel lightweight network architecture, substituting the efficient multi-scale convolution (EMC) module to strengthen the backbone network. The neck network integrates BIG to conduct multi-scale processing, focusing on extracting local spatial detail information and global spatial semantic information. Feature fusion is conducted, and ultimately, the LSH detection head is designed to achieve the lightweight BGLE-YOLO model. The results indicate that the BGLE-YOLO model achieves the same detection accuracy as the benchmark model while significantly reducing the number of parameters and computational demands, successfully balancing accuracy and computational efficiency. The experimental part visually verifies the possibility of optimization of the proposed model. The model’s performance still has room for improvement. Future work will enhance the model’s detection performance and effectively reduce its parameter count and computational efficiency to ensure successful deployment on edge devices and embedded systems for underwater target detection. Additionally, to enhance the robustness of the improved model to better handle more complex marine targets and environmental challenges (such as light interference and fog effects), the detection model will add additional datasets for training.

## Figures and Tables

**Table 1:** Experimental environment parameters.

Training Parameters	Values
Image size	640 × 640
Epochs	300
Batch	32
Workers	8
Learning rate	0.01
Optimizer	SGD
Cache	False
Weight decay factor	0.0005

**Table 2:** Experiments with group normalization/no group normalization are presented on the dataset DUO.

Methods	mAP@0.5 (%)	mAP@0.5:0.95 (%)
YOLOv8n	84.2	65.0
BGLE (w/o GN and DEConv)	83.5	64.9
BGLE (w/GN w/o DEConv)	83.7	64.9
BGLE (w/o GN w/DEConv)	83.9	64.6
BGLE (w/GN and DEConv)	84.2	65.0

**Table 3:** Experiments with group normalization/non-group normalization are presented on the dataset RUOD.

Methods	mAP@0.5 (%)	mAP@0.5:0.95 (%)
YOLOv8n	84.4	60.8
BGLE (w/o GN and DEConv)	82.0	56.2
BGLE (w/GN w/o DEConv)	83.4	58.6
BGLE (w/o GN w/DEConv)	84.0	59.6
BGLE (w/GN and DEConv)	84.1	59.6

**Table 4:** Ablation experiments with DEConv added at different locations on the DUO dataset.

GN	LPC	RPC	mAP@0.5 (%)	mAP@0.5:0.95 (%)	Params (MB)	GFLOPs
			84.2	65.0	3.0	8.1
√			82.7	64.3	1.63	6.2
√	√		83.9	65.1	1.72	6.7
√		√	84.2	65.0	1.63	6.2
√	√	√	83.4	64.7	1.72	6.7

**Table 5:** Ablation experiments with DEConv added at different locations on the RUOD dataset.

GN	LPC	RPC	mAP@0.5 (%)	mAP@0.5:0.95 (%)	Params (MB)	GFLOPs
			84.4	60.8	3.0	8.1
√			84.0	59.6	1.63	6.2
√	√		83.9	59.5	1.72	6.7
√		√	84.1	59.6	1.63	6.2
√	√	√	83.8	59.3	1.72	6.7

**Table 6:** Comparison of BGLE-YOLO components’ performance on the DUO dataset.

Module			Precision (%)	mAP@0.5 (%)	mAP@0.5:0.95 (%)	GFLOPs	Params (MB)
EMC	BIG	LSH					
			82.6	84.2	65.0	8.1	3.0
√			83.0	84.4	65.5	7.6	2.72
	√		86.2	84.8	66.1	7.6	2.14
		√	83.4	84.2	64.9	6.5	2.36
√	√		83.5	84.3	65.7	7.3	2.0
	√	√	85.3	83.9	65.1	6.4	1.77
√	√	√	86.0	84.2	65.0	6.2	1.63

**Table 7:** Performance comparison of BGLE-YOLO components on the RUOD dataset.

Module			Precision (%)	mAP@0.5 (%)	mAP@0.5:0.95 (%)	GFLOPs	Params (MB)
EMC	BIG	LSH					
			85.2	84.4	60.8	8.1	3.0
√			85.4	84.4	60.8	7.6	2.72
	√		85.2	84.3	60.4	7.6	2.14
		√	85.4	84.5	60.3	6.5	2.36
√	√		85.3	84.2	60.1	7.3	2.0
	√	√	84.6	84.0	59.5	6.4	1.77
√	√	√	85.1	84.1	59.6	6.2	1.63

**Table 8:** Comparison of various YOLO network models for underwater detection on the DUO dataset.

Models	Precision (%)	mAP@0.5 (%)	mAP@0.5:0.95 (%)	Params (MB)	GFLOPs
YOLOv5n [36]	84.4	82.4	59.8	1.7	4.1
YOLOv7tiny [37]	86.3	85.1	63.2	6.0	13
YOLOv8n	82.6	84.2	65.0	3.0	8.1
YOLOv10n [38]	83.7	83.0	63.7	2.7	8.2
YOLOv11n [39]	83.7	83.6	64.9	2.6	6.3
BGLE-YOLO	86.0	84.2	65.0	1.6	6.2

**Table 9:** Comparative results of various YOLO family network models for underwater detection on the RUOD dataset.

Models	Precision (%)	mAP@0.5 (%)	mAP@0.5:0.95 (%)	Params (MB)	GFLOPs
YOLOv5n	85.8	83.2	53.9	1.7	4.2
YOLOv7-tiny	85.0	85.2	57.9	6.0	13.1
YOLOv8n	85.2	84.4	60.8	3.0	8.1
YOLOv10n	85.0	83.9	59.9	2.7	8.2
YOLOv11n	85.4	84.7	61.4	2.6	6.3
BGLE-YOLO	85.1	84.1	59.6	1.6	6.2

**Table 10:** Comparative results of different series of network models in underwater exploration under the DUO dataset.

Models	mAP@0.5 (%)	mAP@0.5:0.95 (%)	Params (MB)	GFLOPs
RTMD-Tiny [40]	85.6	66.5	4.8	8.1
DETR-R50 [41]	84.1	62.8	41.5	96.5
YOLOv8	84.2	65.0	3.0	8.1
BGLE-YOLO	84.2	65.0	1.6	6.2

**Table 11:** Comparative results of different series of network models in underwater exploration on the RUOD dataset.

Models	mAP@0.5 (%)	mAP@0.5:0.95 (%)	Params (MB)	GFLOPs
RTMD-Tiny	85.8	62.4	4.88	8
DETR-R50	85.5	59.4	41.55	91.7
YOLOv8	84.4	60.8	3.0	8.1
BGLE-YOLO	84.1	59.6	1.63	6.2

## Data Availability

The data presented in this study are available upon request from the corresponding author. The code cannot be shared due to specific reasons.
